# Salidroside attenuates hypoxia-induced pulmonary arterial smooth muscle cell proliferation and apoptosis resistance by upregulating autophagy through the AMPK-mTOR-ULK1 pathway

**DOI:** 10.1186/s12890-017-0477-4

**Published:** 2017-12-12

**Authors:** Di Gui, Zhimin Cui, Lin Zhang, Chang Yu, Dan Yao, Min Xu, Mayun Chen, Peiliang Wu, Guoping Li, Liangxing Wang, Xiaoying Huang

**Affiliations:** 1 Division of Pulmonary Medicine, The First Affiliated Hospital of Wenzhou Medical University, Key Laboratory of Heart and Lung, Wenzhou, Zhejiang 325000 People’s Republic of China; 2 Department of Invasive Technology, The First Affiliated Hospital of Wenzhou Medical University, Wenzhou, Zhejiang 325000 People’s Republic of China; 3 Department of Respiratory Medicine, Tongde Hospital of Zhejiang Province, Hangzhou, Zhejiang 310013 People’s Republic of China

**Keywords:** PASMCs, Hypoxia, Ampk, mTOR, ULK1, Autophagy

## Abstract

**Background:**

Recent studies have shown that both adenosine monophosphate activated protein kinase (AMPK) and the mammalian target of rapamycin (mTOR) are energy sensors and are related to autophagy. Our recent reports have shown that salidroside can exert protective effects against hypoxia-induced pulmonary arterial smooth muscle cell (PASMC) proliferation and apoptosis resistance through the AMPK pathway. This study aims to explore the relationship among AMPK, mTOR and ULK1 in PASMCs under hypoxic conditions and to investigate whether the protective effects of salidroside are related to the autophagic cell death pathway.

**Methods:**

Rat PASMCs were cultured and divided into five groups: the normoxia, hypoxia, hypoxia + MHY1485 (mTOR agonist), hypoxia + rapamycin (mTOR inhibitor) and hypoxia + salidroside groups. Hypoxic cells were treated as indicated for 24 h. Cell viability was evaluated by the CCK-8 assay. Cell apoptosis was measured by the TUNEL assay. The autophagy flux of PASMCs was evaluated with tandem mRFP-GFP fluorescence microscopy. Autophagosomes were detected by electron microscopy. Protein expression of LC3, p62, AMPK, P-AMPK (Thr 172), P-ULK1 (Ser 555 and Ser 317), mTOR, P-mTOR (Ser 2448), ULK1 and P-ULK1 (Ser 757) was detected by western blot assay.

**Results:**

PASMC proliferation and apoptosis resistance were observed under hypoxic conditions. Autophagy flux, the number of autophagosomes and the LC3II/LC3I ratio were increased in the hypoxia group compared with the normoxia group, whereas p62 expression was decreased. Treatment with rapamycin or salidroside reversed hypoxia-induced PASMC proliferation and apoptosis resistance and further increased autophagy flux, autophagosome levels and the LC3II/LC3I ratio but decreased p62 expression. Treatment with MHY1485 reversed hypoxia-induced PASMC apoptosis resistance and decreased autophagy flux as well as increased autophagosome levels, the LC3II/LC3I ratio and p62 expression. P-AMPK (Thr 172) and P-ULK1 (Ser 555) of the AMPK-ULK1 pathway were increased in the hypoxia group and were further increased in the salidroside group. Rapamycin and MHY1485 had no effect on either P-AMPK (Thr 172) or P-ULK1 (Ser 555). Phosphorylation of ULK1 at serine 317 did not significantly affect the five groups. Furthermore, P-mTOR (Ser 2448) and P-ULK1 (Ser 757) of the AMPK-mTOR-ULK1 pathway were decreased in the hypoxia group and were further decreased in the salidroside group. MHY1485 increased the expression of both P-mTOR(Ser 2448) and P-ULK1(Ser 757), whereas rapamycin had the opposite effect.

**Conclusions:**

Salidroside might inhibit hypoxia-induced PASMC proliferation and reverse apoptosis resistance via the upregulation of autophagy through both the AMPKα1-ULK1 and AMPKα1-mTOR-ULK1 pathways.

## Background

The pathogenesis of pulmonary hypertension (PH) consists of intima lesions, medial vascular remodeling and adventitial remodeling [1]. Pulmonary arterial smooth muscle cells (PASMCs) are the main constituents of medial layer of vessels. Recent studies have revealed that in patients with PH, PASMC hyperplasia plays a key role in medial vascular thickening [2], and their resistance to apoptosis also determines the ultimate fate of PASMCs in PH [1].

Adenosine monophosphate activated protein kinase (AMPK) is an enzyme that can be activated by changes in the AMP/ATP ratio. It can maintain the balance of energy metabolism [3]. AMPK is a heterotrimer that consists of α, β and γ subunits. Both β and γ subunits are regulatory subunits, but the α subunit is a catalytic subunit. The phosphorylation of α subunit at the threonine 172 residue is an essential step for the activation of AMPK [4]. In a variety of diseases, AMPK plays a protective role. It has been reported that metformin inhibits endothelin-1-induced PASMC proliferation via AMPK activation [5]. Additionally, our previous studies have shown that the selective AMPK activator 5′-ami-noimidazole-4-carboxamide ribonucleoside (AICAR) can exert protective effects against hypoxia-induced PASMC proliferation and apoptosis resistance [6, 7]. Therefore, AMPK plays an important role in inhibiting hypoxic pulmonary hypertension (HPH).

Mammalian target of rapamycin (mTOR) is a serine/threonine protein kinase and is also an energy sensor. It is important in the proliferation of PASMCs. Xueping Liu et al. [8] have revealed that mTOR siRNA could inhibit the proliferation of PASMCs under hypoxic conditions. Additionally, Vera P. Krymskaya et al. [9] have revealed that rapamycin, which inhibits mTORC1 signaling, could inhibit chronic hypoxia-induced rat PASMC proliferation.

As energy sensors, AMPK and mTOR have a close relationship. AMPK can downregulate mTOR expression through different pathways [10, 11]. It has been reported that aspirin can help protect against the development of colorectal cancer through the activation of autophagy via upregulating AMPK activity and downregulating mTOR activity [12]. It was reported that the activation of autophagy through the modulation of mTOR-related signaling could result in pro-apoptotic effects on gastric cancer cells [13]. Additionally, in the research of idiopathic pulmonary fibrosis (IPF), Yair Romero et al. [14] found that the persistent activation of mTOR-related pathways decreased the activation of autophagy, which contributed to apoptosis resistance in the IPF fibroblasts. Hypoxia-induced PASMC proliferation possesses several characteristics that are similar to tumor cells [15]. The present study aimed to identify if autophagy might be regulated by the AMPK-mTOR pathway and to confirm if the stimulation of autophagy might exert a protective effect on HPH.

Salidroside has many biological properties, such as cardioprotective, anti-cancer, anti-fatigue and antiviral effects [16–19]. Our previous reports have suggested that salidroside can exert protective effects against hypoxia-induced PASMC hyperproliferation and apoptosis resistance through an AMPKα1-dependent pathway [7]. However, the downstream signaling pathway remains unclear. In this study, we aimed to investigate the downstream signaling pathway of AMPK and reveal whether autophagy plays an essential role in hypoxia-induced PASMC proliferation and apoptosis resistance; additionally, we sought to investigate whether salidroside could attenuate HPH by regulating autophagy through the AMPK-mTOR pathway.

## Methods

### Reagents

Salidroside, 4,6-dimorpholino-N-(4-nitrophenyl)-1,3,5-triazin-2-amine (MHY1485) and collagenase type I were obtained from Sigma (St Louis, MO, USA). Rapamycin was obtained from LC laboratories (Woburn, MA, USA). Dulbecco’s modified Eagle medium (DMEM, high glucose), streptomycin, penicillin G and fetal bovine serums (FBS) were obtained from Gibco BRL (Gaithersburg, MD, USA). The rabbit antibodies against LC3B, GAPDH, P-ULK1 (Ser 757 and Ser 317), mTOR, P-mTOR (Ser 2448) and p62 were obtained from Cell Signaling Technology (Beverly, MA, USA). The rabbit antibody against P-ULK1 (Ser 555) was obtained from US Biological (Swampscott, MA, USA). The rabbit antibody against P-AMPK (Thr 172) was obtained from Abways Technology (Shanghai, China). The rabbit antibody against AMPK was obtained from Abcam (Cambridge, UK). Cell counting kit-8 (CCK-8) was purchased from Dojindo Laboratories (Kumamoto, Japan). The in-situ Cell Death Detection Kit was purchased from Roche Diagnostics (Penzberg, Germany). Tandem monomeric RFP-GFP-tagged LC3 (tfLC3) was purchased from Genechem (Shanghai, China).

### Cell culture and treatment

Rat PASMCs were derived from pulmonary arteries as described previously [6, 7] and were cultured in DMEM supplemented with 100 μg/ml streptomycin, 100 IU/ml penicillin and 10% FBS. Then, cells were divided into five groups: the normoxia (N), hypoxia (H), hypoxia + MHY1485 (mTOR agonist, 2 μmol/L), hypoxia + rapamycin (mTOR inhibitor, 0.5 μmol/L), hypoxia + salidroside (499.5 μmol/L) groups. Hypoxia-treated PASMCs were treated as indicated for 24 h. All hypoxia groups were kept for 24 h in the hypoxia incubator at 37 °C with 5% CO_2_, 5% O_2_ and 90% N_2_, whereas the normoxia group was kept in a normal incubator at 37 °C with 21% O_2_, 5% CO_2_ and 74% N_2_.

### Cell viability assay

Cell viability was determined by the CCK-8 assay. PASMCs were seeded in 96-well microplates at a concentration of 1 × 10^4^ cells/well. After they were preincubated in complete medium at 37 °C in 21% O_2_ and 5%CO_2_ for 24 h, PASMCs were pretreated with salidroside, rapamycin or MHY1485 before exposure to hypoxia. Cell growth was observed under a microscope before the CCK-8 assay. After 24 h of hypoxia, CCK-8 was added to the cells at a concentration of 10 μl/well for 2 h. A microplate reader was used to determine the absorbance at 450 nm.

### Cell apoptosis detection

After pretreatment, cells that adhered to the cover slips were fixed with fresh 4% paraformaldehyde for 1 h at 15 °C-25 °C. Cells were incubated with 3% H_2_O_2_ for 10 min at 15 °C-25 °C. Then, 0.1% Triton X-100 was incubated with the cells for 15 min at room temperature. Both anti-goat serum and 5% BSA were used to block non-specific binding. An in-situ Cell Death Detection Kit was used according to the manufacturer’s instructions. DAB and hematoxylin were used for cell staining. Light microscopy was used to observe the cover slips. Five randomly selected fields from each cover slip were analyzed to determine the percentage of terminal deoxynucleotidyl (TUNEL)-positive cells.

### Autophagy flux detected by tandem mRFP-GFP fluorescence microscopy

Cells were seeded onto 6-well microplates at a concentration of 5 × 10^4^ cells/well. After preincubation in complete medium at 37 °C in 21% O_2_ and 5%CO_2_ for 24 h, the cells were transfected with tfLC3 according to the manufacturer’s instructions to monitor autophagy flux. Eight hours after transfection, the cells were washed with PBS; next, complete culture medium was added to the cells. At 48 h after transfection, the cells were treated with salidroside, rapamycin or MHY1485. Cells were incubated in the hypoxia chamber for another 24 h. Finally, the samples were examined under a fluorescence microscope (Nikon, Tokyo, Japan).

### Autophagosome detection of rat PASMCs

Cells were fixed with both 2.5% glutaraldehyde and 1% osmic acid in sequence and then stained with 1% uranyl acetate. Acetone was used to dehydrate the cells, and then the cells were embedded in epoxy resin 812. Ultra-microtome slices V (Sweden) were used to cut the fixed cells into ultrathin sections. The number of autophagosomes in the cells was evaluated with Hitachi H-600 transmission electron microscopy (Hitachi, Japan).

### Detection of autophagy-related proteins LC3B and p62 by western blotting analysis

Cells were harvested and incubated with ice-cold RIPA buffer containing PMSF. Then, the samples were centrifuged at 4 °C at 12000 rpm for 15 min. The Bradford method was used to quantify the protein concentrations. A total of 40 μg of protein from each group was separated using 12% SDS-PAGE. The proteins were transferred to PVDF membranes and blocked with 5% skimmed milk. Specific primary antibodies against LC3B (1:1000, 2775S) and p62 (1:1000, 5114S) were used to detect the proteins. GAPDH (1:1000, 5174S) was used as an internal control. Horseradish peroxidase-conjugated secondary antibodies were incubated with the proteins at a 1:10,000 dilution. Quantity one-4.6.2 software (Bio-Rad Laboratories, Hercules, CA, USA) was used to quantify the density of immunoblots after the detection of immunoreactive bands with BeyoECL Plus reagents (Beyotime, China).

### The AMPK-ULK1 pathway-related proteins AMPKα1, P-AMPKα1 (Thr 172), P-ULK1 (ser 555) and P-ULK1 (ser 317) were detected by western blotting analysis

A total of 40 μg of protein from each group was separated using 8% SDS-PAGE. Specific primary antibodies against AMPKα1 (1:2000, ab32047), phosphorylated AMPKα1 (1:2000, CY5556), phosphorylated ULK1 (Ser 555, 1:1000, U1500-70F) and phosphorylated ULK1 (Ser 317, 1:1000, 12753S) were used to detect the proteins. The experimental procedures for western blotting analysis were performed as described above.

### The mTOR-ULK1 pathway-related proteins mTOR, P-mTOR, ULK1 and P-ULK1 (ser 757) were detected by western blotting analysis

A total of 40 μg of protein from each group was separated using 8% SDS-PAGE. Specific primary antibodies against mTOR (1:1000, 2983S), phosphorylated mTOR (1:1000, 5536S), ULK1 (1:1000, 8054 s) and phosphorylated ULK1 (Ser 757, 1:1000, 14202S) were used to detect the proteins. The experimental procedures for western blotting analysis were performed as described above.

### Statistical analysis

The results were expressed as the mean ± standard deviation (SD). Statistical significance was determined with one-way ANOVA followed by the least significant difference (LSD) test. A value of *P* < 0.05 was considered to be statistically significant. All calculations were performed by SPSS version 21.0.

## Results

### Autophagy was involved in hypoxia-induced PASMC proliferation and apoptosis resistance

**Fig. 1:**
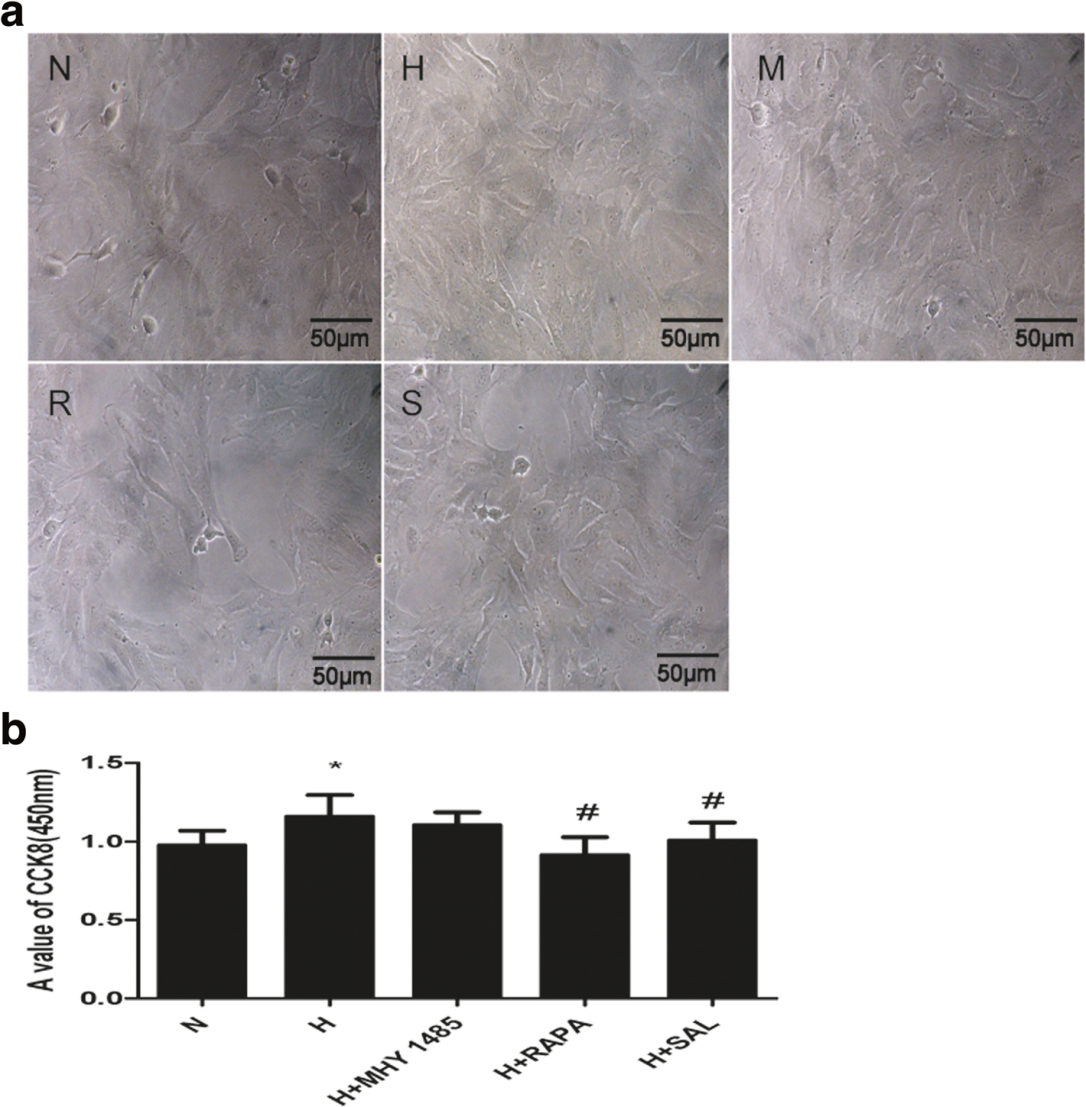
Autophagy is involved in hypoxia-induced PASMC proliferation. **a** Cell density in each group as viewed under a microscope. **b** CCK-8 values in each group. *p < 0.05 vs. the N group; and #*p* < 0.05 vs. the H group

**Fig. 2:**
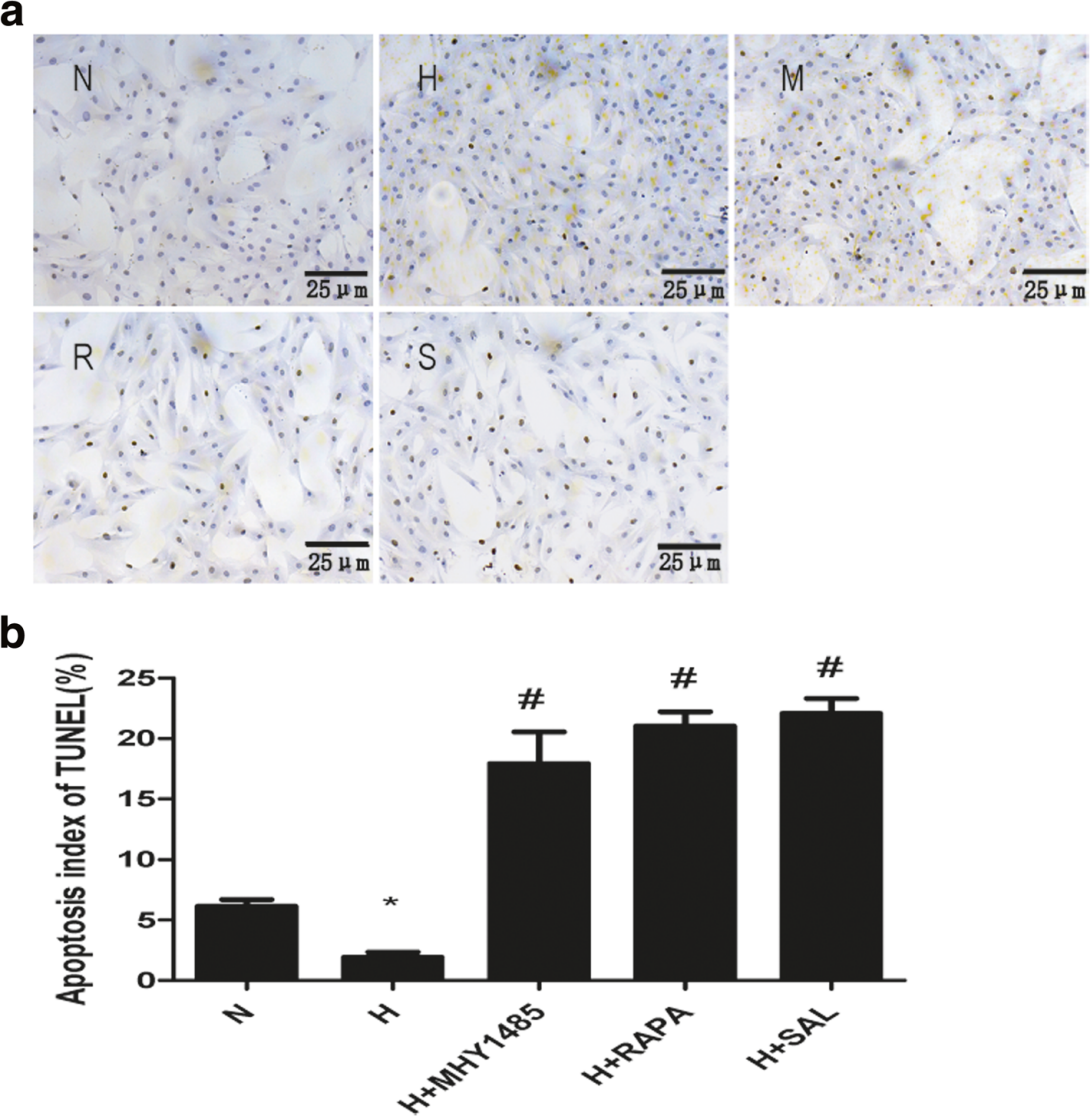
Autophagy is involved in hypoxia-induced PASMC apoptosis resistance. **a** Images of cell apoptosis in each group as determined by the TUNEL assay. **b** Quantitative analysis of the cell apoptosis index by TUNEL assay. *p < 0.05 vs. the N group; and #*p* < 0.05 vs. the H group

**Fig. 3:**
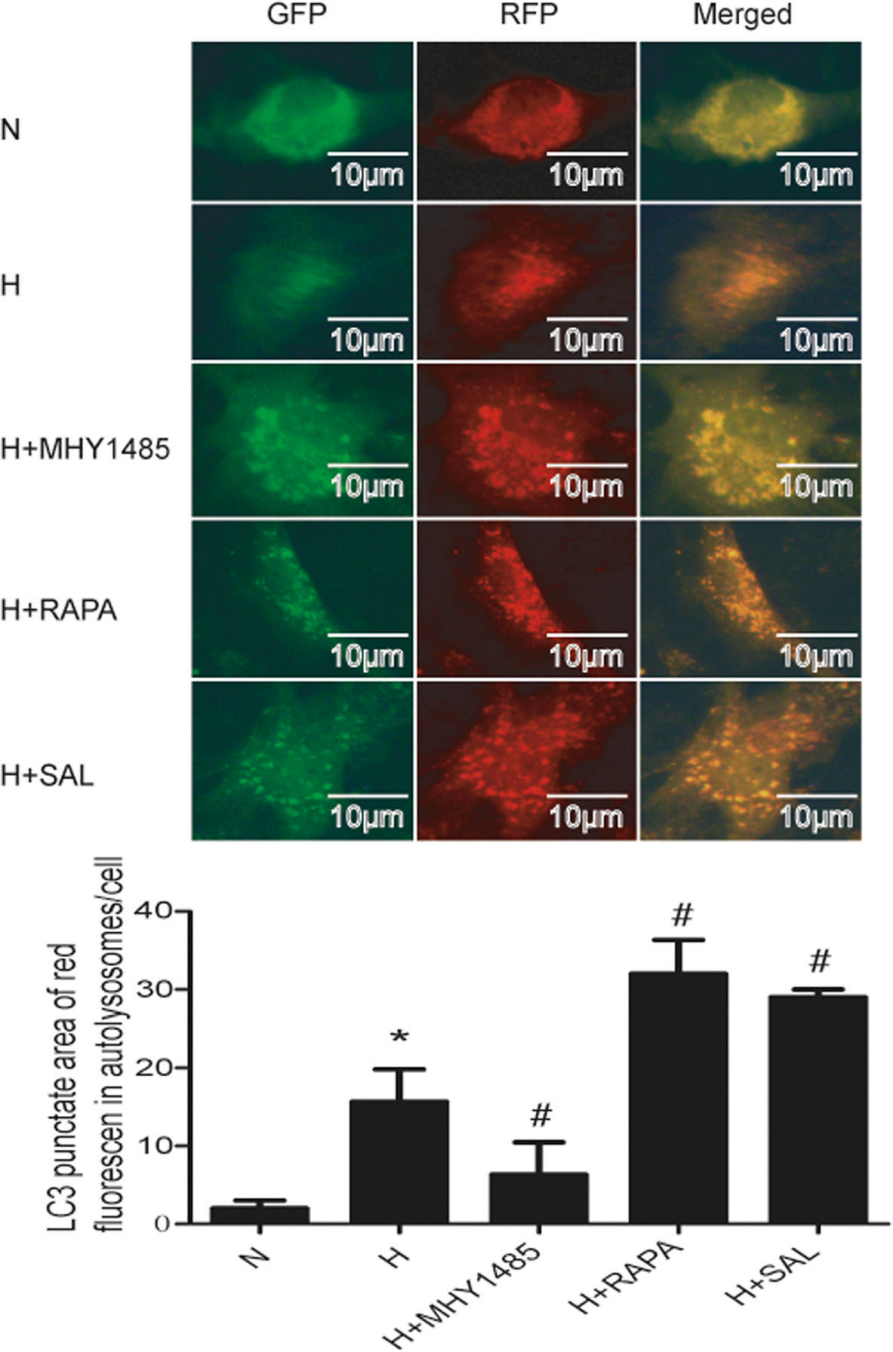
Enhanced autophagy flux may be involved in the effects of salidroside on PASMC proliferation and apoptosis resistance under hypoxic conditions. After transfection with tfLC3, PASMCs were exposed to hypoxia and treated with MHY1485, rapamycin or salidroside; next, the cells were observed under a fluorescence microscope. *p < 0.05 vs. the N group; and #*p* < 0.05 vs. the H group

**Fig. 4:**
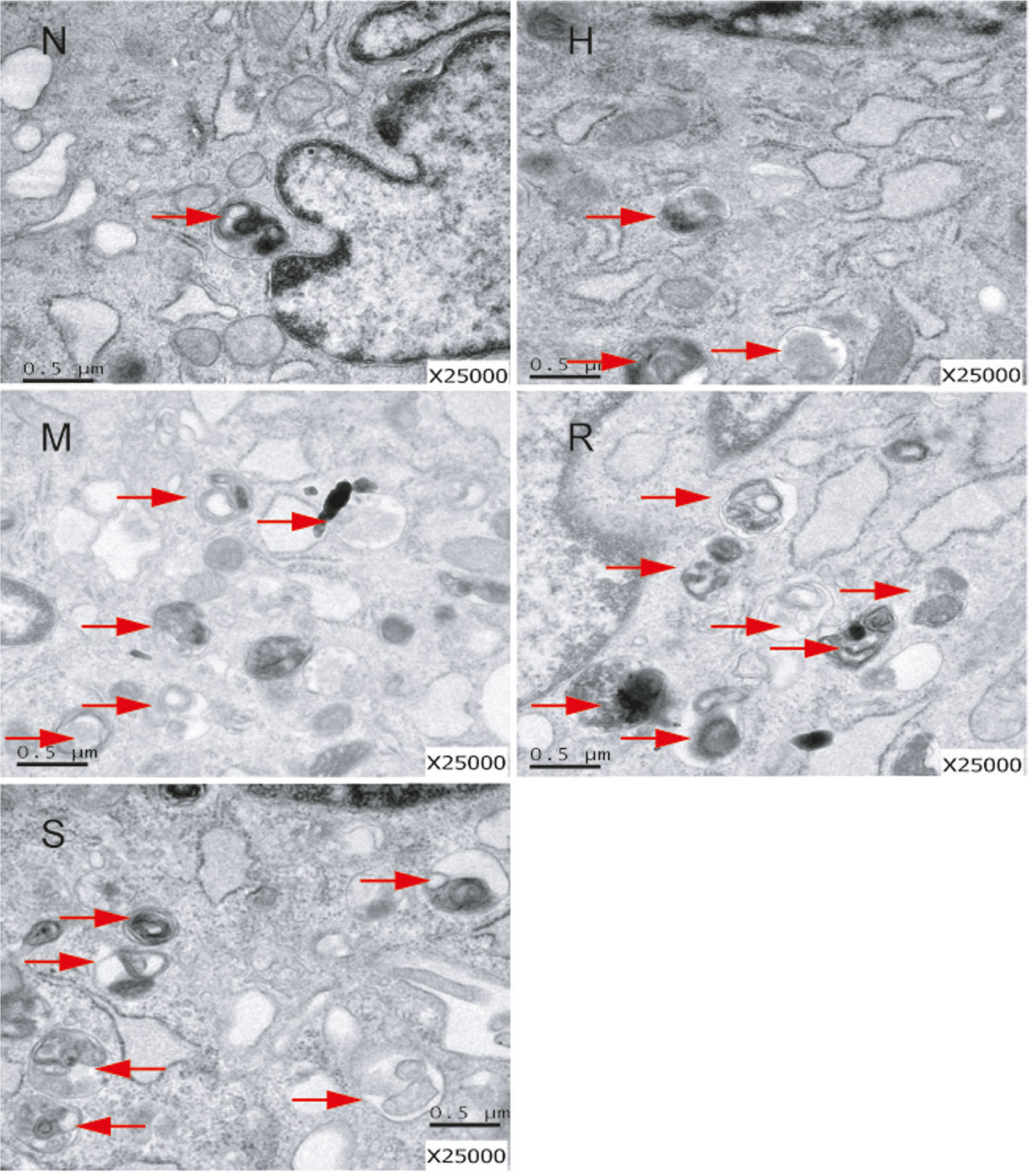
Increased numbers of autophagosomes may be related to salidroside-induced decreases in the proliferation and increases in the apoptosis of PASMCs under hypoxic conditions. Images of autophagosomes in PASMCs of each group acquired by Hitachi H-600 transmission electron microscopy. The control group exhibited less autophagosomes than the hypoxia group. The salidroside group exhibit increased numbers of autophagosomes than the hypoxia group. Red arrow: autophagosomes

**Fig. 5:**
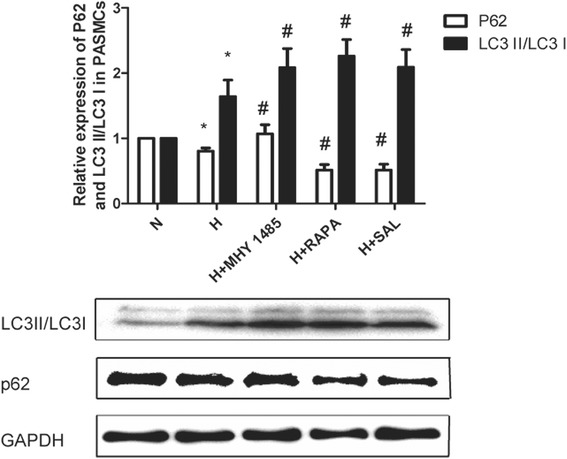
Increased autophagy may be related to the salidroside-induced attenuation of PASMC proliferation and apoptosis resistance under hypoxic conditions. Images and quantitative analysis of p62 expression and the LC3II/LC3I ratio in PASMCs by western blot assay. GAPDH was used as an internal control. *p < 0.05 vs. the N group; and #*p* < 0.05 vs. the H group

PASMCs were treated with hypoxia with or without rapamycin or MHY1485 to demonstrate the role of autophagy in hypoxia-induced PASMC proliferation and apoptosis resistance. As shown in Fig. 1a and b, the density of PASMCs under the microscope in the H group was greater than that in the N group. Additionally, the viability of hypoxia-treated PASMCs was obviously increased (*P* < 0.05). The density and viability of cells treated with rapamycin were reduced (*P* < 0.05), whereas MHY1485 had no effect on cell density and viability (*P* > 0.05). As shown in Fig. 2a and b, the apoptosis index of PASMCs under hypoxic conditions was obviously decreased (*P* < 0.05). The apoptosis index of PASMCs treated with rapamycin or MHY1485 was increased (*P* < 0.05). Meanwhile, the number of autophagosomes, the ratio of LC3II to LC3I and autophagic flux were increased and the expression of p62 was decreased in cells treated with hypoxia. These changes were further enhanced in PASMCs treated with rapamycin (Figs. 3, 4 and 5). Therefore, we infer that hypoxia could enhance autophagy, which can further be enhanced by rapamycin. In PASMCs treated with MHY1485, although the number of autophagosomes and the ratio of LC3II to LC3I were increased, the autophagic flux of PASMCs was decreased, and the expression of p62 was increased (Figs. 3, 4 and 5). Therefore, we concluded that MHY1485 could inhibit autophagy. These results indicate that the increase in autophagy under hypoxic conditions might compensatory, and further enhanced autophagy may decrease PASMC proliferation and increase PASMCs apoptosis. The detailed mechanisms underlying the decrease in autophagy, increase in the apoptosis index, and lack of effects on proliferation following MHY1485 treatment in hypoxic PASMCs were investigated next.

### Enhanced autophagy flux may underlie the effects of salidroside on PASMC proliferation and apoptosis resistance under hypoxic conditions

To confirm autophagy activation following different interventions, we transfected tfLC3 into PASMCs (Fig. 3). Since the GFP signal is sensitive to acidic conditions, this form of LC3 displays only red fluorescence when located in autolysosomes. Increased red spots were observed in hypoxic cells. Furthermore, an increased number of red spots were observed in cells treated with rapamycin and salidroside. However, both green and red spots were observed in cells treated with MHY1485. These data revealed that hypoxia enhanced the autophagy flux of cells, which was further enhanced by rapamycin and salidroside. However, MHY1485 weakened the autophagy flux of PASMCs.

### Increased numbers of autophagosomes may be related to the salidroside-induced decrease in the proliferation and increase in the apoptosis of PASMCs under hypoxic conditions

To confirm the number of autophagosomes in PASMCs after different interventions, transmission electron microscopy was performed (Fig. 4). The number of autophagosomes were increased in cells under hypoxic conditions. The number of autophagosomes were further increased in cells treated with salidroside, rapamycin and MHY1485. These results together with the autophagy flux analysis indicated that salidroside increased the number of autophagosomes by activating autophagy. However, MHY1485 increased the number of autophagosomes through suppressing lysosomal fusion.

### Increased autophagy may be related to the salidroside-induced attenuation of PASMC proliferation and apoptosis resistance under hypoxic conditions

To confirm autophagy activation following different interventions, protein levels of LC3 and p62 were detected by western blotting analysis (Fig. 5). The ratio of LC3II to LC3I was increased and p62 expression was diminished in the hypoxia group (*P* < 0.05). In both the rapamycin and salidroside groups, changes in the ratio of LC3II to LC3I and p62 expression were further enhanced (*P* < 0.05). However, both the ratio of LC3II to LC3I and p62 expression were increased in the MHY1485 group compared to the hypoxia group (*P* < 0.05). These data together with autophagy flux and autophagosome detection revealed that rapamycin and salidroside further increases autophagy compared to hypoxia, which reverses PASMC proliferation and apoptosis resistance (Figs. 1a, b and 2a, b). However, increased levels of autophagosomes in the MHY1485 group resulted in increased LC3II expression, which may be related to the reversal of PASMC proliferation and apoptosis resistance.

### The AMPK-ULK1 (ser 555) pathway, which regulates autophagy, is upregulated by salidroside treatment in PASMCs

**Fig. 6:**
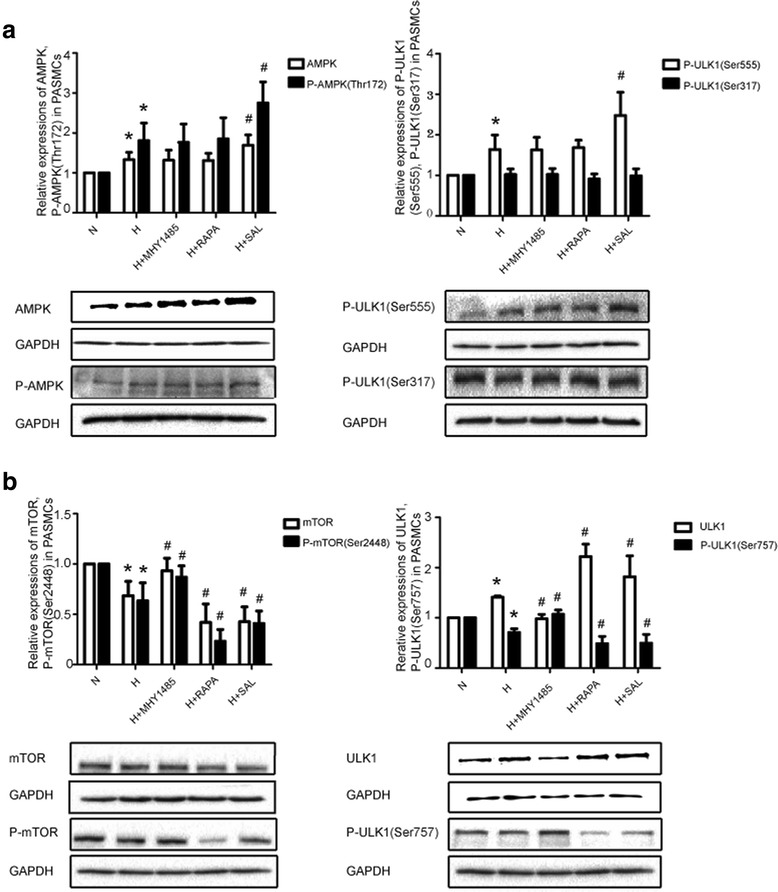
The AMPK-ULK1 (Ser555) pathway is upregulated and The mTOR-ULK1 (Ser757) pathway is downregulated by salidroside in PASMCs. **a** Images and quantitative analysis of AMPKα1, phosphorylated AMPKα1, and phosphorylated ULK1 (Ser555, 317) in PASMCs by western blot assay. **b** Images and quantitative analysis of mTOR, phosphorylated mTOR, ULK1 and phosphorylated ULK1 (Ser 757) in PASMCs by western blot assay. GAPDH was used as an internal control. *p < 0.05 vs. the N group; and #*p* < 0.05 vs. the H group

**Fig. 7:**
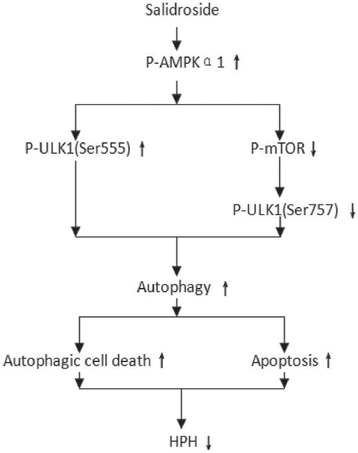
The signaling pathways of this experiment. Salidroside exerted protective effects against hypoxic PASMCs via the upregulation of autophagy through both the AMPK-ULK1 (Ser 555) and AMPK-mTOR-ULK1 (Ser 757) pathways

To confirm whether the increase in autophagy induced by salidroside is dependent on the regulation of the AMPK-ULK1 pathways, cells were treated with hypoxia and salidroside. The levels of AMPK, phosphorylated AMPK and phosphorylated ULK1 (Ser 555) were upregulated in the hypoxia group. The changes in AMPK, phosphorylated AMPK and phosphorylated ULK1 (Ser 555) expression were further enhanced in the salidroside group compared to the hypoxia group (*P* < 0.05). To further confirm whether the role of salidroside is mTOR dependent, cells were treated with the mTOR inhibitor rapamycin and the mTOR agonist MHY1485. The expression of these proteins in cells treated by MHY1485 and rapamycin was similar to that in the hypoxia group (*P* > 0.05). The phosphorylation of ULK1 at serine 317 had no significant effect on the five groups (*P* > 0.05) (Fig. 6a). These data revealed that salidroside can exert a protective effect through the AMPK-ULK1 (Ser 555) pathway (Fig. 7).

### The mTOR-ULK1 (ser 757) pathway, which regulates autophagy, was downregulated by salidroside in PASMCs

To confirm whether the increase in autophagy induced by salidroside was also dependent on the regulation of the mTOR-ULK1 pathway, mTOR, P-mTOR, ULK1 and P-ULK1 (S757) protein levels were detected (Fig. 6b). Protein expression of mTOR, phosphorylated mTOR and phosphorylated ULK1 (Ser 757) was decreased in the hypoxia group (*P* < 0.05). Changes in mTOR, phosphorylated mTOR and phosphorylated ULK1 (Ser 757) expression were further enhanced in the salidroside and rapamycin groups compared to the hypoxia group (*P* < 0.05). Protein expression levels of mTOR, phosphorylated mTOR and phosphorylated ULK1 (Ser 757) were increased in the MHY1485 group compared to the hypoxia group (*P* < 0.05). The expression of ULK1 contrasts that of P-ULK1 (Ser 757). These data revealed that salidroside can exert a protective effect through the mTOR-ULK1 (Ser 757) pathway (Fig. 7).

## Discussion

Autophagy is the process by which cellular components are self-degraded. Portions of cytosol or organelles are sequestered by double-membrane autophagosomes, which fuse with lysosomes and are degraded by resident hydrolases [20]. Autophagy can be divided into three main types according to the different delivery pathways of cargo to the lysosome: macroautophagy, microautophagy and chaperone-mediated autophagy (CMA) [21]. Macroautophagy is the most widely studied process among the three main forms of autophagy, and the present study focuses on it. The process of autophagy consists of induction, vesicle nucleation, membrane elongation, autophagosome formation, autophagosome maturation/cargo assimilation, autophagosome lysosome fusion, autolysosomal acidification, substrate degradation and recycling [22]. Induction is initiated by the activation of the ATG1 complex [23]. UNC-51-like kinase 1 (ULK1) and 2 are the mammalian homologs of ATG1 [24]. Most importantly, ULK1 constantly associates with ATG13, and thus, ULK1 is the key regulatory protein for mammalian autophagy initiation [25].

HPH is one of the main complications of chronic obstructive pulmonary diseases, which is associated with pulmonary vascular remodeling caused by chronic hypoxia. Endothelial cell dysfunction, excessive contraction of blood vessels and smooth muscle cell hyperplasia are all associated with pulmonary vascular remodeling [26]. Therefore, inhibition of PASMC hyperproliferation and apoptosis resistance may be an efficient therapeutic strategy for HPH.

In a previous study, we revealed that 5-Aminoimidazole-4-carboxamide ribonucleotide (AICAR), an agonist of AMPK, decreases proliferation and increases the apoptosis index of hypoxic PASMCs [6]. Additional studies have revealed that salidroside has the same effect as AICAR [7]. However, its molecular mechanism has not been fully investigated.

The result of the CCK-8 assay showed that hypoxia increases the viability of PASMCs, and salidroside reverses the effect of hypoxia. Rapamycin, which enhances autophagy in cells, obviously inhibited the viability of PASMCs. As reported by Juliana Navarro-Yepes et al. [27], the autophagy-induced excessive degradation of essential cellular components may cause autophagic cell death. In the present study, MHY1485, an agonist of mTOR, slightly decreased the viability of PASMCs. Yeon Ja Choi et al. [28] revealed that MHY1485 not only inhibits autophagy by activating mTOR but also suppresses lysosomal fusion. In addition, Daniel J. Klionsky et al. [29] revealed that the reasons for the accumulation of autophagosomes include upregulating autophagy induction and downregulating autophagosome degradation. In our study, although MHY1485 increased autophagosome and LC3BII levels, it inhibited autophagy flux, which concurred with the findings of Yeon Ja Choi et al. [28]. Although decreased autophagy flux may be related to an increase in the proliferation of PASMCs, increased LC3B could exert opposite effects [30]. This explains why MHY1485 did not have an effect on PASMC viability under hypoxic conditions. Altogether, we concluded that autophagy may be increased as a compensatory response to hypoxia and that further enhancements in autophagy may lead to a decrease in cell proliferation.

Using the TUNEL assay, the present study showed that hypoxia decreased the apoptosis index of PASMCs. Salidroside obviously reversed the effect of hypoxia. Rapamycin, which induces autophagy, also increased the apoptosis index of PASMCs under hypoxic conditions. As reported by Jacob M. Gump et al. [31], enhanced autophagy might selectively promote Fas-mediated apoptotic cell death. MHY1485 also increased the apoptosis index of PASMCs under hypoxic conditions, which may also be attributed to its pharmacological action. MHY1485 increased autophagosome and LC3B levels in a manner that is similar to the induction of autophagy. Furthermore, Shi Chen [32] reported that the accumulation of autophagosomes may aggravate alveolar macrophage apoptosis. Therefore, we infer that enhanced autophagy may increase PASMC apoptosis in HPH.

In a previous study [7], we demonstrated that salidroside can exert protective effects on PASMCs under hypoxic conditions through an AMPK-dependent pathway. Dmitry A.Goncharov et al. [33] revealed that AMPK could reverse PASMC proliferation by downregulating mTORC1 signaling. mTOR is an autophagy-associated protein, and it can inhibit autophagy through the phosphorylation of ULK1 [25, 34]. Moreover, previous studies have revealed that AMPK can enhance autophagy by directly phosphorylating the serine sites of ULK1, which is the protein responsible for autophagy induction [34, 35]. Therefore, AMPK can phosphorylate ULK1 through both direct and indirect pathways. Altogether, we infer that salidroside may exert protective effects on PASMCs under hypoxic conditions through both the AMPK-ULK1 and AMPK-mTOR-ULK1 pathways.

In this study, we first investigated the AMPK-ULK1 pathway. We applied MHY1485, an mTOR activator, or rapamycin, an mTOR inhibitor. Then, we determined if the changes in the level of phosphorylated ULK1 in the hypoxia and salidroside group were mTOR dependent or mTOR independent by comparing their results with that of the MHY1485- and rapamycin-treated groups. Joungmok Kim et al. [34] revealed that AMPK can phosphorylate ULK1 at serine 317 and serine 777 in HEK293 cells. Additionally, the study by Daniel F. Egan et al. [35] revealed that AMPK can phosphorylate ULK1 at the serine 555, serine 467, serine 627 and threonine 574 residues in HEK293T cells. Therefore, we choose to detect ULK1, a key protein of the mTOR independent pathway, at the serine 555 and serine 317 residues. The levels of AMPK, phosphorylated AMPK and phosphorylated ULK1 (Ser 555) were upregulated under hypoxic conditions, and salidroside further enhanced the changes in the levels of these proteins. However, the expression of these proteins in PASMCs treated with MHY1485 or rapamycin was similar to that in the hypoxia group. The phosphorylation of ULK1 at serine 317 did not lead to significant changes in the five groups. Therefore, we concluded that the AMPK-ULK1 (Ser 555) pathway is involved in the regulation of autophagy by salidroside in PASMCs. In a future study, we will further investigate other residues of ULK1 (Fig. 7).

The mTOR-ULK1 pathway was also investigated. Recent studies have revealed that AMPK can inhibit the activity of mTOR [10, 11]. On the other hand, mTOR can inhibit autophagy through the phosphorylation of ULK1 at serine 757 [25, 34]. In our study, the levels of mTOR, phosphorylated mTOR and phosphorylated ULK1 (Ser 757) were downregulated in the hypoxia group. Salidroside and rapamycin further enhanced the changes in the expression of these proteins, and MHY1485 had the opposite effect. The expression of total ULK1 is opposite of that of P-ULK1 (Ser 757). Therefore, we concluded that the mTOR-ULK1 (Ser757) pathway was also involved in the regulation of autophagy by salidroside in PASMCs (Fig. 7).

### Summary

In our research, autophagy flux, autophagosome levels and the ratio of LC3II to LC3I were increased and p62 expression was decreased in PASMCs under hypoxic conditions; additionally, salidroside further enhanced the abovementioned changes and attenuated hypoxia-induced pulmonary arterial smooth muscle cell proliferation and apoptosis resistance. We concluded that the increase in autophagy in hypoxic PASMCs was a compensatory response and that salidroside could further enhance autophagy. Enhanced autophagy induced by salidroside through both the AMPKα1-ULK1 (Ser 555) and AMPKα1-mTOR-ULK1 (Ser 757) pathways could decrease cell proliferation and increase cell apoptosis in PASMCs under hypoxic conditions.

## Conclusions

In conclusion, our study demonstrated that salidroside effectively inhibits PASMC proliferation and apoptosis resistance under hypoxic conditions by upregulating autophagy through both the AMPKα1-ULK1 (Ser 555) and AMPKα1-mTOR-ULK1 (Ser 757) pathways. This study provides novel evidence of the protective effects of salidroside against HPH.

## Abbreviations

AMPK: adenosine monophosphate activated protein kinase
ATG: Autophagy-related gene
DMEM: Dulbecco’s modified Eagle medium
HPH: hypoxic pulmonary hypertension
IPAH: idiopathic pulmonary arterial hypertension
IPF: idiopathic pulmonary fibrosis
mTOR: mammalian target of rapamycin
PASMCs: pulmonary arterial smooth muscle cells
PH: pulmonary hypertension
Ser: serine
tfLC3: tandem monomeric RFP-GFP-tagged LC3
Thr: threonine
TSC: tuberous sclerosis proteins; and raptor: regulatory-associated protein of mTOR
ULK: UNC-51-like kinase
